# Spontaneous Coronary Reperfusion in STEMI After Pre‐Hospital Loading Dose of Triple Antithrombotic Therapy

**DOI:** 10.1002/clc.70418

**Published:** 2026-07-18

**Authors:** Pierre Alain Meunier, Lea Juenin, Lionel Moulis, François Roubille, Pierre Robert, Jean Christophe Macia, Jean‐Michel Berdeu, Matthieu Steinecker, Benoit Lattuca, Laurent Schmutz, Luc Cornillet, Guillaume Cayla, Florence Leclercq

**Affiliations:** ^1^ Department of Cardiology CHU Montpellier Montpellier France; ^2^ Department of Cardiology CHU Nîmes Nîmes France; ^3^ Epidemiological and Clinical Research Unit, CHU Montpellier Montpellier France

**Keywords:** out‐of‐ hospital cardiac arrest, P2Y12 inhibitors, primary PCI, spontaneous reperfusion, STEMI, TIMI 3 flow

## Abstract

**Background:**

To evaluate the incidence and prognosis of spontaneous coronary reperfusion in STEMI using pre‐hospital loading dose of P2Y12 inhibitors.

**Methods:**

This prospective bi‐centric study included STEMI patients who referred for primary PCI from June 1, to November 1, 2022. All patients received before hospital admission loading dose of aspirin, heparin and P2Y_12_ inhibitor. The primary outcome was incidence of spontaneous coronary reperfusion (TIMI 3 flow) before primary PCI. Major in‐hospital and 1‐year events were secondary outcomes.

**Results:**

Within the study period, 263 patients were admitted with ongoing STEMI and 49 of them (18.6%) had spontaneous coronary reperfusion with no significant difference between loading dose of ticagrelor (*n* = 226; 85.9%) or clopidogrel (*n* = 27, 10.3%) and TIMI 3 flow (*n* = 41; 18.1% vs. *n* = 7; 25.9%, *p* = 0.53; respectively). Peak of T‐hs troponin (*p* < 0.001) and CRP (*p* = 0.03) were significantly lower in the TIMI 3 group. In multivariate analysis, out‐of‐ hospital cardiac arrests were observed twice as often in the TIMI 3 group (16.33 vs. 7.01%; *p* = 0.044). There was no significant difference between the two groups regarding major in‐hospital (*p* = 0.69) or 1‐year major events (*p* = 0.20).

**Conclusion:**

Spontaneous reperfusion at admission was observed in about 20% of STEMI patients who received a pre‐hospital loading dose of antithrombotic therapy including P2Y_12_ inhibitors, and was associated with markers of decrease of infarct size. The significant increase of pre‐admission cardiac arrests observed in the TIMI 3 group could be specifically explored as it might be a concern regarding future pre‐hospital reperfusion therapy strategies.

## Summary

- Spontaneous coronary reperfusion is observed in nearly 20% of a contemporary population of patients with STEMI using pre‐hospital loading dose of triple antithrombotic therapy and is significantly associated with improvement of biological parameters of infarct size.
- The twice as often incidence of pre‐hospital cardiac arrest observed (*p* = 0.044) has to be further explored.

## Graphical abstract

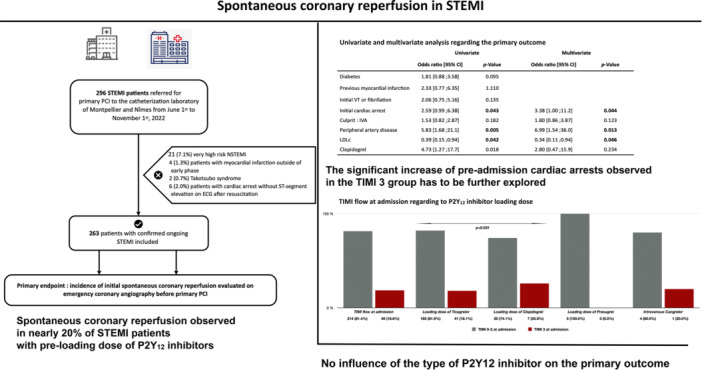

**Abbreviations**
ACS: acute coronary syndrome
BARC: bleeding Academic Research Consortium
CRP: C reactive protein
GP: general practitioner
LVEF: left ventricular ejection fraction
MACE: major cardiovascular events
PCI: percutaneous coronary intervention
STEMI: ST‐segment elevation myocardial infarction
TIMI: thrombolysis in myocardial infarction

## 1 Introduction

The rapid and complete restoration of coronary flow is a key issue in the management of ST‐segment elevation myocardial infarction (STEMI) [1, 2, 3, 4, 5]. When possible, primary percutaneous coronary intervention (PCI) associated with antithrombotic drugs is the preferred reperfusion strategy [6, 7, 8, 9]. In daily practice, some patients may achieve complete spontaneous reopening of the culprit artery at admission in the catheterization laboratory before undergoing any mechanical reperfusion therapy, which is called « spontaneous reperfusion » [10]. Spontaneous reperfusion is defined as initial TIMI 3 flow (as classified by the Thrombolysis in Myocardial Infarction trial) of the culprit coronary artery observed on diagnostic coronary angiography before PCI [10, 11, 12, 13]. It is associated with improved outcomes in several and often old studies [10, 11, 12, 13, 14, 15] or without systematic use of a prehospital antithrombotic strategy including new P2Y_12_ inhibitors (ticagrelor and prasugrel mainly) at first medical contact, according to 2017 guidelines (grade I) [8] and also possible in the 2023 guidelines (grade IIb) [9].

The objectives of this study were to report contemporary incidence, clinical characteristics, and prognostic impact of spontaneous coronary reperfusion of STEMI patients admitted for primary PCI using systematic pre‐hospital loading doses of P2Y_12_ inhibitors.

## 2 Materials and Methods

### 2.1 Study Design and Population

This bi‐center, observational and prospective study was conducted between June 1 and November 1, 2022 in the cardiology department of Montpellier and Nîmes University Hospitals, France. All consecutive patients referred to the catheterization laboratory for primary PCI during the study period were screened. We included all patients with STEMI defined by the European Society of Cardiology and receiving, according to 2017 guidelines, preloading dose of aspirin, heparin and P2Y_12_ inhibitor as soon as possible in the pre‐hospital phase [8]. The study included patients in the early phase of STEMI (pain lasting less than 12 h) or with evolved STEMI (pain lasting between 12 and 48 h) with ongoing symptoms suggestive of ischemia, including persistent chest pain, hemodynamic instability, or life‐threatening arrhythmias.

Exclusion criteria were linked to patients referred for coronary angiography after thrombolysis and myocardial infarction outside of acute phase as previously defined.

The study was approved by the ethical committee of the hospital (IRB ID 202100821) and was conducted in accordance with the 1964 Helsinki declaration and subsequent revisions. Informed oral and written consent were obtained for all patients.

The study was registered on the clinical trial website (NCT 04881552).

### 2.2 Pre‐Hospital Antithrombotic Therapy

Triple antithrombotic therapy (i.e., loading dose of aspirin, heparin, and P2Y12 inhibitor) was given at first medical contact for all patients in the pre‐hospital phase by the emergency medical staff according to regional protocols defined in European guidelines. Aspirin was administered at a dose of 500 mg intravenously. According to guidelines [8, 9], clopidogrel (600 mg, oral) was usually reserved to contraindications of others more potent P2Y_12_ inhibitors (ticagrelor and prasugrel) related to bleeding risk including long‐term anticoagulant therapy. Ticagrelor was mainly used in our centers (180 mg, sublingual) while prasugrel (60 mg, oral) was more rarely used related to local emergency medical assistance service (*Service d'Aide Médicale Urgente*) practice. Intra venous (IV) cangrelor (30 mcg/kg bolus immediately followed by a 4 mcg/kg/min IV infusion) was proposed when patients presenting vomiting after administration of ticagrelor or to patients unable to absorb oral agents. Regarding anticoagulation therapy, IV enoxaparin (0.5 g/kg), was used in first line, as proposed in guidelines [8].

### 2.3 Coronary Angiography Procedure

Coronary angiography was performed under local anesthesia. Right radial approach was the default strategy, otherwise left radial or transfemoral access was used. Mechanical thrombectomy (ASAP aspiration catheter, Merit) was performed for thrombotic burden lesions with indication left at the discretion of the interventional cardiologist. New generation drug‐eluting stents (Orsiro, Biotronik; XIENCE Sierra, Abbott; Ultimaster Tansei, Terumo; Resolute Onyx, Medtronic) were implanted using a routine method. Only the infarct‐related artery (IRA) was treated during the initial intervention according to guidelines [9]. For patients with multivessel disease, staged revascularization was usually performed before hospital discharge.

Spontaneous reperfusion was defined as TIMI flow grade 3 observed on emergency coronary angiography before primary PCI of the culprit coronary artery. According to previous reports, TIMI grade 3 flow was defined as opacification of the coronary artery within three cardiac cycles and when antegrade flow into the bed distal to the obstruction occurred as promptly as antegrade flow into the bed proximal to the obstruction, with clearance of contrast material from the involved bed was as rapid as clearance from an uninvolved bed in the same vessel or the opposite artery [12]. A TIMI 0 (complete occlusion), TIMI 1 (no distal coronary perfusion), or TIMI 2 flow (perfusion of entire artery with delayed flow) was considered as an occluded artery [10, 11, 12, 13, 14].

### 2.4 Study Outcomes

The primary outcome was the incidence of initial spontaneous coronary TIMI flow grade 3, evaluated on emergency coronary angiography before primary PCI.

Secondary endpoints were assessed in the global population and in the groups with or without TIMI 3 flow and included: (1) In‐hospital major clinical events including death, cardiogenic shock, cardiac arrest, ventricular arrhythmias, mechanical complications, heart failure (HF), myocardial re‐infarction, stroke and serious hemorrhagic event (Bleeding Academic Research Consortium [BARC] ≥ 3a); (2) in‐hospital biological settings related to infarct size including dosage of pic T‐hs troponin and C‐reactive protein (CRP); (3) echocardiographic evaluation before hospital discharge of left ventricular ejection fraction (LVEF); (4) length of hospital stay; (5) major cardiovascular events (MACE) including death, new ACS, or re‐hospitalization for heart failure or stroke at 1‐year follow‐up.

### 2.5 Data Collection and Follow‐up

In‐hospital clinical, biologic, and echocardiographic data were collected during the hospital stay for all patients. Data at 1‐year follow‐up were obtained from medical records and by calling patient, general practitioner (GP), or cardiologist.

All the data were collected and analyzed according to the CNIL MR‐003 reference.

### 2.6 Statistical Analysis

We expected that the incidence of TIMI 3 flow at admission (primary end point) would be about 20% according to previous reports [10, 11, 12, 13, 14].

Categorical data were reported as counts and percentages. Continuous data were reported as mean ± standard deviation for normally distributed data. Comparisons used the chi‐squared test or Fisher's exact test for categorical variables and Student's test or the Mann–Whitney–Wilcoxon test, as appropriate, for continuous variables. For predictors of initial TIMI flow, a multivariate logistic regression was performed. All potential predictors with a *p*‐value < 0.20 in univariate were entered in an initial model. Variables were selected through a backward elimination procedure based on the best Akaike Information Criteria to obtain the final model. A two‐tailed *p* < 0.05 was considered statistically significant. All statistics were performed using EasyMedStat (www.easymedstat.com) or R 4.4.

## 3 Results

### 3.1 Study Population (Table 1)

**Figure 1:**
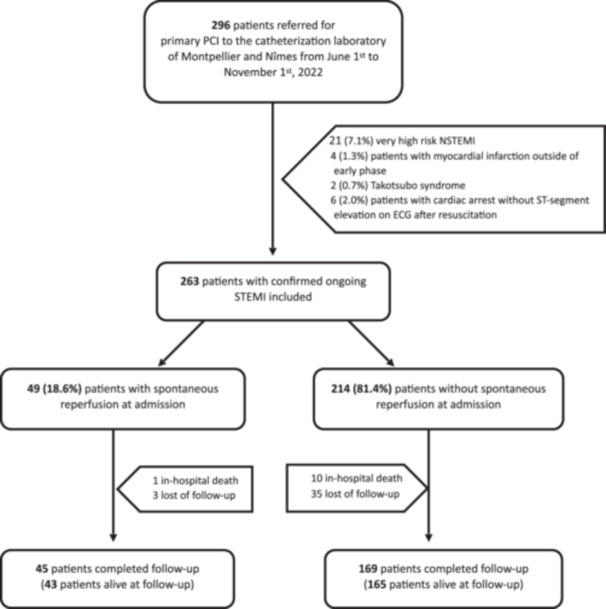
Study flow chart.

During the study period, 263 patients who referred to the catheterization laboratory for primary PCI were confirmed to have STEMI and were included in the study (Figure 1). Mean age was 63 ± 13 years old and 207 were male (78.7%). Hypertension and active smoking were the main cardiovascular risk factors. Anterior STEMI was observed in 106 patients (40.3%) while inferior STEMI was diagnosed in 112 patients (46.2%). Pre‐infarction angina pectoris was diagnosed in 63 patients (24.0%) and Initial presentation with out‐of‐hospital cardiac arrest (OHCA) was observed in 23 patients (8.7%).

**Table 1:** Patients characteristics at admission.

	Global population (*n* = 263)	TIMI 0–2 at admission (*n* = 214)	TIMI 3 at admission (*n* = 49)	*p*
Demographics				
Age (years), mean	63 (±13)	62 (±13)	65 (±14)	0.18
Male	207 (78.7%)	171 (79.9%)	36 (73.5%)	0.42
BMI (kg/m^2^), mean	27 (±4)	27 (±4)	26 (±5)	0.20
Cardiovascular risk factors				
Obesity (BMI > 30 kg/m^2^)	45 (21.7%)	40 (23.5%)	5 (13.5%)	0.27
Hypertension	102 (38.8%)	84 (39.5%)	18 (36.7%)	0.87
Diabetes	57 (21.7%)	42 (19.6%)	15 (30.6%)	0.14
Dyslipidaemia	88 (33.5%)	74 (34.6%)	14 (28.6%)	0.52
Active smoking	107 (40.7%)	89 (41.6%)	18 (36.7%)	0.64
Family history of CAD	57 (21.8%)	49 (23.0%)	8 (16.3%)	0.41
Comorbidities				
Previous CAD	40 (15.2%)	31 (14.5%)	9 (18.4%)	0.51
Previous myocardial infarction	18 (6.8%)	12 (5.7%)	6 (12.2%)	0.12
Peripheral artery disease	11 (4.2%)	5 (2.3%)	6 (12.2%)	0.007
Stroke	8 (3.0%)	6 (2.8%)	2 (4.1%)	0.65
Atrial fibrillation	12 (4.6%)	9 (4.2%)	3 (6.1%)	0.47
Chronic kidney failure				
GFR < 60 mL/min/1.73 m^2^	15 (5.7%)	12 (5.6%)	3 (6.1%)	> 0.99
GFR ≤ 30 mL/min/1.73 m2	3 (1.1%)	0 (0.0%)	3 (6.1%)	0.006
Current treatment				
Anti‐platelet therapy				
Aspirin	45 (17.1%)	34 (16.0%)	11 (22.4%)	0.30
Clopidogrel	10 (3.8%)	5 (2.4%)	5 (10.2%)	0.023
Both	43 (16.3%)	31 (14.6%)	12 (24.5%)	0.13
Anticoagulation therapy				
Vitamin K antagonist	2 (0.8%)	1 (0.5%)	1 (2.0%)	0.34
DOA therapy	12 (4.6%)	9 (4.2%)	3 (6.1%)	0.47
Beta‐blockers	45 (17.1%)	31 (14.6%)	14 (28.6%)	0.034
Statins	41 (15.6%)	31 (14.6%)	10 (20.4%)	0.38
Presentation				
Preexisting angina pectoris	63 (24.0%)	48 (22.4%)	15 (30.6%)	0.31
Initial cardiac arrest	23 (8.7%)	15 (7.0%)	8 (16.3%)	0.049
Initial 3° atrioventricular block	12 (4.6%)	10 (4.7%)	2 (4.1%)	> 0.99
Initial ventricular tachycardia or fibrillation	23 (8.7%)	16 (7.5%)	7 (14.3%)	0.16
Localization of ST‐segment elevation on ECG				
Anterior	106 (40.3%)	82 (38.3%)	24 (49.0%)	0.23
Inferior	112 (42.6%)	97 (45.3%)	15 (30.6%)	0.09
Posterior	7 (2.7%)	5 (2.3%)	2 (4.1%)	0.62
Lateral	38 (14.4%)	30 (14.0%)	8 (16.3%)	0.66
Initial laboratory tests				
Creatinine at admission (µmol/L), mean	92 (±59)	86 (±25)	98 (±93)	0.25
Hemoglobin at admission (g/dL), mean	14 (±1.5)	14 (±1.5)	14 (±1.5)	0.82
LDL‐cholesterol (g/L)	1.20 (±0.38)	1.27 (±0.39)	1.14 (±0.36)	0.045
HbA1c (%), mean	6.09 (±1.28)	6.07 (±1.32)	6.1 (±1.24)	0.06

*Note:* Values are mean (±DS) or *n* (%).

Abbreviations: BMI, body mass index; CAD, coronary artery disease; DOA, direct oral anticoagulation; GFR, glomerular filtration rate estimated with CKD‐EPI equation; HbA1c, glycosylated hemoglobin; LDL, low density lipoprotein.

### 3.2 Primary End Point

**Figure 2:**
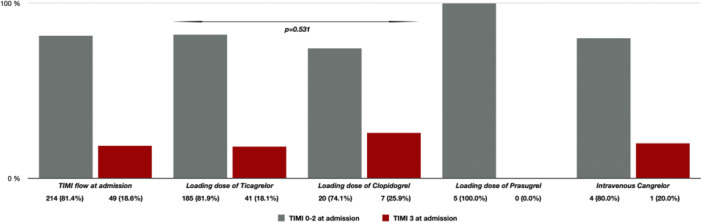
TIMI flow at admission and according to P2Y_12_ inhibitor loading dose.

Spontaneous reperfusion on initial angiography (TIMI 3 flow, primary end‐point) was observed in 49 out of 263 patients (18.6%) while 214 patients (81.4%) have occluded coronary artery (TIMI 0, 1, or 2) at admission (Figure 1, graphical abstract). A pre‐hospital loading dose of ticagrelor was given in 226 patients (85.9%), while the others receiving clopidogrel or more rarely prasugrel or cangrelor (Figure 2).

Delay between symptoms onset to primary PCI was similar between the two groups with respectively 304 min (±360) in TIMI 3 group (*p* = 0.66) and 270 min (± 203) in TIMI 0–2 group (Table 2). Considering procedural characteristics, we observed higher thrombotic burden lesions in the TIMI 0–2 group (*p* = 0.007) and use of mechanical thrombectomy and balloon pre‐dilatation were also more frequently used in this group of patients (respectively *p* = 0.003 and *p* = 0.049) (Table 2).

**Table 2:** Antithrombotic therapy and angiographic data at admission and after primary PCI in the two groups of patients.

	TIMI 0–2 at admission (*n* = 214)	TIMI 3 at admission (*n* = 49)	*p*
Pre‐treatment			
Loading dose of P2Y12 inhibitor			
Clopidogrel	20 (9.3%)	7 (14.3%)	0.30
Ticagrelor	185 (86.5%)	41 (83.7%)	0.65
Prasugrel	5 (2.3%)	0 (0.0%)	0.59
Cangrelor	4 (1.9%)	1 (2.0%)	0.43
Enoxaparin	210 (98.1%)	45 (91.8%)	0.042
Unfractionned heparin	4 (1.9%)	4 (8.2%)	0.042
Delays (min), mean			
Symptoms onset to primary PCI	270 (±203)	304 (±360)	0.66
Culprit artery			
Left anterior descending artery	91 (42.5%)	26 (53.1%)	0.24
Diagonal artery	11 (5.1%)	2 (4.1%)	> 0.99
Circumflex artery	23 (10.8%)	5 (10.2%)	> 0.99
Left obtuse marginal artery	11 (5.1%)	3 (6.1%)	0.73
Right coronary artery	75 (35.1%)	13 (26.5%)	0.33
Ramus intermedius artery	4 (1.9%)	0 (0.0%)	> 0.99
High thrombus burden lesion	127 (59.3%)	18 (36.7%)	0.007
Multivessel disease	108 (50.5%)	32 (65.3%)	0.09
Treatment during angiography			
Mechanical thrombectomy	65 (30.4%)	4 (8.2%)	0.003
Glycoprotein IIb/IIIa inhibitors	59 (27.6%)	13 (26.5%)	> 0.99
Pre‐dilation	119 (55.6%)	19 (38.8%)	0.049
PCI with stent(s)	197 (92.1%)	46 (93.9%)	> 0.99
Post‐dilation	52 (24.3%)	19 (38.8%)	0.06
TIMI flow grade at the end of the procedure			0.72
TIMI 3	199 (93.0%)	48 (98.0%)	
TIMI 2	6 (2.8%)	0 (0.0%)	
TIMI 1	4 (1.9%)	1 (2.0%)	
TIMI 0	5 (2.3%)	0 (0.0%)	

Abbreviation: PCI, percutaneous coronary intervention.

### 3.3 TIMI 3 Flow Incidence in Univariate and Multivariate Analysis

No significant difference was observed between loading dose of ticagrelor or clopidogrel regarding TIMI flow at admission (81.9% and 74.1% for TIMI 0–2 vs. 18.1% and 25.9% for TIMI 3, *p*‐value = 0.53) (Figure 2).

Regarding patients characteristics and in univariate analysis (Table 1), there were significantly more patients in the TIMI 3 group with history of peripheral artery disease (*p* = 0.007) or severe renal disease (*p* = 0.006), lower LDL level (0.045), pre‐hospital medical therapy with clopidogrel (*p* = 0.023), or beta‐blockers (*p* = 0.034). Considering initial presentation, OHCA was observed twice as often in the TIMI 3 group (*n* = 15, 7% vs. *n* = 8, 16.3%, *p* = 0.049) as well as ventricular tachycardia or fibrillation before admission, although non‐ significant for this later (*n* = 16, 7.5% vs. *n* = 7, 14.3%, *p* = 0.16). These patients received cangrelor (*n* = 5) when they were intubated at admission, the others received ticagrelor after resolved cardiac arrest before admission (*n* = 18). Multivariate analysis is presented in Table 3 and confirmed only previous PAD, lower LDL as associated with TIMI 3 flow, as initial presentation with cardiac arrest (confidence interval [CI]: 95%, odds ratio [OR]: 3.38, [1.00; 11.2], *p* = 0.044).

**Table 3:** Univariate and multivariate analysis regarding the primary outcome.

	Univariate		Multivariate	
	Odds ratio [95% CI]	*p‐*value	Odds ratio [95% CI]	*p*‐value
Diabetes	1.81 [0.88; 3.58]	0.095		
Previous myocardial infarction	2.33 [0.77; 6.35]	1.110		
Initial VT or fibrillation	2.06 [0.75; 5.16]	0.135		
Initial cardiac arrest	2.59 [0.99; 6.38]	**0.043**	3.38 [1.00; 11.2]	**0.044**
Culprit: IVA	1.53 [0.82; 2.87]	0.182	1.80 [0.86; 3.87]	0.123
Peripheral artery disease	5.83 [1.68; 21.1]	**0.005**	6.99 [1.54; 36.0]	**0.013**
LDLc (every 10 mg/dL)	0.39 [0.15; 0.94]	**0.042**	0.34 [0.11; 0.94]	**0.046**
Clopidogrel	4.73 [1.27; 17.7]	0.018	2.80 [0.47; 15.9]	0.234

*Note:* Bold values indicate statistically significant.

### 3.4 In‐Hospital Outcomes (Table 4)

Killip class I at admission in ICU was observed in all patients with TIMI 3 flow and 199 out of 214 (94.3%) patients with TIMI 0–2 on initial angiography (*p* = 0.3). Peaks of troponin (*p* < 0.001) and CRP (*p* = 0.031) were significantly lower in the TIMI 3 group.

The electrocardiogram performed at admission in the intensive care unit (ICU) highlighted less incidence of Q wave in the TIMI 3 group (*p* = 0.018).

There was no significant difference regarding in‐hospital events in the TIMI 3 group compared to TIMI 0–2 group (*p* = 0.69). However statistically no significant, in‐hospital death was more than twice as frequent in this later group (*n* = 1, 2.0% vs. *n* = 10, 4.7%; *p* = 0.69). Duration of hospitalization was similar between the two groups.

**Table 4:** In‐hospital evolution in the two groups of patients.

	TIMI 0–2 at admission (*n* = 214)	TIMI 3 at admission (*n* = 49)	*p*
At admission to the ICU			
Chest pain	186 (91.6%)	44 (95.6%)	0.62
Killip class			0.3
I	199 (94.3%)	49 (100.0%)	
II	5 (2.4%)	0 (0.0%)	
III	3 (1.4%)	0 (0.0%)	
IV	4 (1.9%)	0 (0.0%)	
Electrocardiogram			
Q wave	**126 (59.7%)**	**19 (39.6%)**	**0.018**
Regression of ST‐segment elevation	150 (71.4%)	38 (77.6%)	0.49
Echocardiographic characteristics			
LVEF (%), mean	48 (±9)	50 (±8)	0.11
Moderate impaired LVEF (40‐50%)	121 (57.1%)	27 (55.1%)	0.93
Impaired LVEF (< 40%)	25 (11.9%)	7 (14.3%)	0.63
Right ventricular dysfunction	18 (8.5%)	1 (2.0%)	0.23
Pericardial effusion	1 (0.5%)	1 (2.0%)	0.34
Laboratory test			
Peak of troponin (ng/L)	**5351.2 (**± **6483.1)**	**2334.7 (**± **3013.3)**	**< 0.001**
Peak of CRP (mg/L)	**57.0 (**± **84.6)**	**38.9 (**± **57.6)**	**0.031**
Major in‐hospital events	42 (19.6%)	8 (16.3%)	0.69
Ventricular arrhythmias	5 (2.3%)	0 (0.0%)	0.59
Conduction disorders	10 (4.7%)	0 (0.0%)	0.22
Cardiac arrest	8 (3.7%)	1 (2.0%)	> 0.99
Congestive heart failure	17 (7.9%)	5 (10.2%)	0.57
Cardiogenic shock	8 (3.7%)	0 (0.0%)	0.36
Tamponade	1 (0.5%)	0 (0.0%)	> 0.99
Stroke	2 (0.9%)	1 (2.0%)	0.46
Intraventricular thrombus	5 (2.3%)	1 (2.0%)	> 0.99
Mechanical complications			
Free wall rupture	1 (0.5%)	1 (2.0%)	0.34
Ventricular septal defect	0 (0.0%)	1 (2.0%)	0.19
Stent thrombosis	0 (0.0%)	0 (0.0%)	1
All cause in‐hospital death	10 (4.7%)	1 (2.0%)	0.69
Duration of hospitalization (days), mean	6 (±4)	6 (±4)	0.38

*Note:* Bold values indicate statistically significant.

Abbreviations: CRP, C‐reactive protein; LVEF, left ventricular ejection fraction.

### 3.5 Follow‐up (Table 5)

At 1‐year, 35 patients were lost to follow‐up in the TIMI 0–2 group, and three patients in the TIMI 3 group. Six new patients died, four patients in the TIMI 0–2 group and two in the TIMI 3 group (*p* = 0.61). No significant difference was highlighted regarding major adverse events between the two groups (*p* = 0.20) but mean LVEF was higher in the TIMI 3 group (56 ± 13 vs. 54 ± 11; *p* = 0.032).

**Table 5:** One year follow‐up in the two groups of patients.

	TIMI 0–2 at admission (*n* = 214)	TIMI 3 at admission (*n* = 49)	*p*
Major carviovascular events at 1 year follow (excluding in‐hospital events)	**32 (15.0%)**	**11 (22.5%)**	0.20
Re‐hospitalization for cardiologic reason	**27 (16.9%)**	**10 (22.7%)**	0.38
Angina pectoris	8 (5.0%)	4 (9.1%)	0.29
Heart failure	3 (1.9%)	0 (0.0%)	> 0.99
Acute coronary syndrome (ACS)	1 (0.6%)	2 (4.6%)	0.12
Myocardial ischemia demonstrated on functional non‐invasive tests	4 (2.5%)	3 (6.8%)	0.17
Coronary angiography	18 (11.3%)	8 (18.2%)	0.31
PCI	8 (5.0%)	4 (9.1%)	0.29
Initial culprit coronary artery	4 (2.5%)	4 (9.1%)	0.07
Stent thrombosis	0 (0.0%)	1 (2.3%)	0.22
In‐stent restenosis	2 (1.3%)	3 (6.8%)	0.07
Stroke	1 (0.6%)	0 (0.0%)	> 0.99
Serious bleeding event (≥ BARC3)	2 (1.3%)	1 (2.3%)	0.52
All cause death at 1‐year follow‐up (excluding in‐hospital death)	4 (2.5%)	2 (4.6%)	0.61
LEVF (%), mean	**54 (**±**11)**	**56 (**±**13)**	**0.032**

*Note:* Bold values indicate statistically significant.

Abbreviations: LEVF, left ventricular ejection fraction; PCI, percutaneous coronary intervention.

## 4 Discussion

This prospective bi‐centric observational study aimed to evaluate the incidence and the prognostic of spontaneous reperfusion of the culprit coronary artery in a contemporary population of patients admitted for primary PCI of STEMI and who received a systematic pre‐hospital loading dose of anti‐thrombotic therapy including P2Y_12_ inhibitors. The main results were: (1) Spontaneous reperfusion was observed in nearly 20% of patients, similarly to previous studies without use of pre‐hospital loading dose of P2Y12 inhibitors; (2) The delay between symptoms onset to primary PCI or the type P2Y_12_ inhibitor did not impact incidence of TIMI 3 flow at admission; (3) A significant increase of incidence of pre‐hospital cardiac arrest was highlighted in the spontaneous reperfusion group; (4) Spontaneous reperfusion was significantly associated with improvement of prognostic factors relating to infarct size but with no difference regarding MACE at 1‐year follow‐up.

### 4.1 TIMI 3 Flow Incidence at Baseline Angiography

Previous studies have shown that the incidence of spontaneous reperfusion of STEMI admitted in the cathlab was about 15%–30% [10, 11, 12, 13, 14, 15, 16, 17, 18]. Spontaneous reperfusion was also reported without any therapy in about 20% of STEMI with possible determinants including platelet reactivity and faster endogenous fibrinolysis [19]. Many studies were undergoing without using P2Y_12_ inhibitors as a pre‐hospital strategy [10, 11, 14]. Wang et al. [18], using clopidogrel as pre‐treatment, observed 26% of TIMI 3 flow at admission in a retrospective analysis including 519 patients. Montalescot et al. [20], in the prospective and randomized ATLANTIC study compared as a secondary endpoint, the incidence of TIMI flow grade 3 at admission when ticagrelor was used in a pre‐ or in an in‐hospital strategy and showed no significant difference (17.4% vs. 16.9%, *p* = 0.82). Our results are consistent with these reports but also with those of older studies that do not use pre‐hospital loading dose of P2Y12 inhibitors [10, 11, 14]. Unlike the ATLANTIC study, all our patients received pre‐hospital loading dose of P2Y_12_ inhibitors. Interestingly, nor the delay of loading dose nor the type of P2Y_12_ inhibitor (mainly ticagrelor vs. clopidogrel) impacted the incidence of TIMI 3 flow at admission as observed in the ATLANTIC study by Montalescot et al. [20]. In the HELP‐PCI trial, heparin administration at first medical contact resulted in higher rates of TIMI 3 flow on initial angiography (pre‐PCI) against a pre‐catheterization injection in the cathlab with no significant difference in bleeding [21]. This strategy of pre‐hospital heparin administration was systematic in our study using a triple anti‐thrombotic strategy. These results, even non‐comparative, support the hypothesis that the pre‐ hospital loading dose of P2Y_12_ inhibitors in STEMI do not impact coronary reopening before PCI and that the benefit of P2Y_12_ inhibitors in STEMI, even with new generation molecules, is probably related to decrease of ischemic events including stent thrombosis during the hospital phase and thereafter [20, 21, 22, 23].

Therefore, the pre‐treatment with a P2Y_12_ inhibitor was reconsidered in the 2023 ESC guidelines for the management of acute coronary syndromes, and was reclassified from I to IIb, regarding no scientific evidence of benefits [9]. Interestingly, in the COMPARE CRUSH trial, crushed prasugrel did not improve TIMI 3 flow at first angiography compared with integral prasugrel, both of which were administered as a 60 mg load in the ambulance prior to PCI and despite observed lower platelet reactivity in the crushed prasugrel arm [24]. This result argued also against benefit of systematic pre‐hospital and “as soon as possible” loading dose of P2Y_12_ inhibitor in STEMI, but rather to an in‐hospital strategy after angiographic evaluation of coronary anatomy, similarly as recommended in NSTEMI [9].

### 4.2 In‐Hospital Course

Our study, even unpowered regarding in‐hospital clinical events, confirmed that spontaneous coronary reperfusion has prognostic impact in STEMI regarding clinical and biological factors associated with smaller infarct size. Interestingly, the success of PCI was high in the two groups (93% vs. 98%, *p* = 0.72) and may not explain the differences regarding infarct size parameters.

Previous reports [11, 12, 13, 14, 15, 16], most of them performed before coming up of new P2Y_12_ inhibitors, showed that patients with spontaneous reperfusion tended to have all the features of improved myocardial salvage including less cardiac enzyme release, higher left ventricular ejection fraction, fewer in‐hospital complications, and a better outcome than patients without spontaneous reperfusion. Interestingly, we observed no ventricular arrhythmias, no conduction disorders and no cardiogenic shock during hospitalization in the TIMI 3 group and, even no significant, there was more than twice less in‐hospital deaths in this group. Lastly, all patients in the TIMI 3 group were in Killip class 1 at admission, in accordance with previous studies [13, 14, 25]. In our study, delays between symptoms onset to primary PCI were not significantly different between the two groups so the differences in in‐hospital evolution identified are not related to difference in delays of admission or delays of therapy.

### 4.3 TIMI 3 Flow and Patient's Profile

In univariate analysis, history of PAD, severe kidney disease, previous clopidogrel or beta‐blockers therapy or lower LDL level were as significantly associated with TIMI 3 flow at admission. Multivariate analysis showed only PAD (*p* = 0.013) and lower LDL at admission (*p* = 0.046) as associated with TIMI 3 flow. These results could suggest that a vascular history, perhaps partly related to previous antithrombotic and statin therapy may impact spontaneous coronary reperfusion in STEMI. Wang et al. [18] showed that patients with spontaneous reperfusion were younger in age, with culprit lesions mostly located in the left anterior descending artery. Similarly to others reports, we did not find significant difference regarding the culprit coronary artery in the TIMI 3 group [18, 20]. Only one study showed that patients with spontaneous reperfusion were slightly older [25], but the relation between age and spontaneous reperfusion remains unclear. Two others studies found that pre‐infarction angina was associated with the occurrence of spontaneous reperfusion [13, 18], but we did not highlight a difference about this criterion in our patients.

### 4.4 Pre‐Hospital Cardiac Arrest and TIMI 3 Flow

In multivariate analysis, initial presentation with OHCA (*p* = 0.044) before hospital admission were observed twice as often in the TIMI 3 group. Although not statistically significant, severe ventricular arrhythmia were also twice as common diagnosed in this group of patients (*p* = 0.16). Coronary reperfusion syndrome including bradycardia or tachycardia may then have occurred more frequently in the TIMI 3 group before hospital admission [26]. It was found in subsequent laboratory experiments that ventricular fibrillation may occur more frequently after reperfusion than after coronary artery occlusion [26, 27]. Indeed, myocardial reperfusion may cause profound electrophysiological alteration and reperfusion‐induced arrhythmias were reported as an underestimated cause of sudden cardiac death [27, 28]. This observation could be a concern regarding protocols currently evaluated self‐administration of antithrombotic therapy in subjects with a recent history of acute myocardial infarction, like SOS AMI study, currently in progress (NCT 20214069).

### 4.5 Follow‐Up at 1‐Year

At 1‐year follow‐up, no significant differences were highlighted regarding major events although we observed a trend to more ACS and symptomatic stent thrombosis in the TIMI 3 group, but without statistical significance. LVEF was significantly higher in the TIMI 3 group, and usually correlates with long‐term prognostic. The 1‐year mortality was similar between the two groups which was in accordance with previous studies [20, 21], showing significant relation between spontaneous reperfusion and improved survival at 30 days, but not at 1 year, which can be related to impact of others prognostic parameters or too short follow‐up. Differences of prognostic associated with TIMI 3 flow observed between early and more recent studies may also be related to differences in antithrombotic regimens, the use of more potent P2Y12 inhibitors, advances in revascularization strategies, or improvements in post‐PCI care [10, 11, 12, 13, 14, 15].

## 5 Study Limitations

The first limitation of the study is related to the relatively small number of patients and events, with consequently lack of power which makes interpretation of secondary endpoints with caution. The second limitation is the relatively short, 5‐month study duration and the single‐region design. Third, the inclusion of patients presenting up to 48 h after symptom onset should also be noted. While this increases generalizability, it could also influence the observed rate of spontaneous reperfusion, as a longer timeframe allows for more natural clot dissolution. Fourth, the definition of reperfusion with an angiographic criterion while no reflow phenomena is not taken into account but we used strict definition of angiographic TIMI 3 flow. Fifth, patients lost to follow‐up, particularly in the TIMI 0–2 group could have potentially influence results. Lastly, we evaluate P2Y_12_ inhibitors but mainly ticagrelor and clopidogrel, and these results cannot be extended to prasugrel or cangrelor.

## 6 Conclusion

This prospective contemporary study shows an incidence of about 20% of STEMI patients admitted for primary PCI, with angiographic evidence of spontaneous reperfusion (TIMI 3 flow). This incidence is the same that observed in older studies despite the use of a pre‐hospital antithrombotic strategy using mainly new generation P2Y_12_ inhibitors and suggests, despite non randomized, lack of influence of this strategy on the reperfusion rate of the culprit artery. TIMI 3 flow at admission remains, as previously described, associated with lower risk profile of STEMI regarding clinical and biological parameters of infarct size and probably to better long‐ term follow‐up. Improvement of pre‐hospital coronary reperfusion could be an important issue in future STEMI therapy whereas the increased risk of pre‐hospital cardiac arrest observed in our study in spontaneous reperfused patients, could be specifically explored.

## Author Contributions

Substantial contributions to the conception or design of the work or the acquisition, analysis or interpretation of data for the work by Florence Leclercq, Pierre Alain Meunier, Lea Juenin, Lionel Moulis. Drafting the work or revising it critically for important intellectual content by Florence Leclercq, Pierre Alain Meunier, Lionel Moulis, Pierre Robert, Jean Christophe Macia, and Jean‐Michel Berdeu. Final approval of the version to be published by Florence Leclercq, Lionel Moulis, François Roubille, Guillaume Cayla, Matthieu Steinecker, Benoit Lattuca, Laurent Schmutz, and Guillaume Cayla. Agreement to be accountable for all aspects of the work in ensuring that questions related to the accuracy or integrity of any part of the work are appropriately investigated and resolved by Florence Leclercq, Lionel Moulis, Guillaume Cayla, François Roubille, Guillaume Cayla, Matthieu Steinecker, Benoit Lattuca, and Luc Cornillet.

## Conflicts of Interest

Florence Leclercq has received research Grants or consultant fees from Boehringer Ingelheim, Edwards Lifesciences, Medtronic. Guillaume Cayla has received research Grants to the Institution or Consulting/Lecture Fees from Amgen, AstraZeneca, Bayer, Boehringer Ingelheim, Biotronik, Bristol‐Myers Squibb, Daiichi‐Sankyo, Eli‐Lilly, Medtronic, MSD, Pfizer, Sanofi‐Aventis. Pierre Robert has received research grants from Edwards Lifesciences. Benoit Lattuca has received research grants from Biotronik, Boston Scientific, Daiichi‐Sankyo, Fédération Française de Cardiologie and Institute of CardioMetabolism and Nutrition; consultant fees from Daiichi‐Sankyo and Eli Lilly; and lecture fees from AstraZeneca, Medtronic and Novartis. The remaining authors declare no conflicts of interest.

## Data Availability

The research data associated with a paper are available, at University Hospital of Montpellier. The data that support the findings of this study are available on request from the corresponding author. The data are not publicly available due to privacy or ethical restrictions.
